# Feasibility of Comparing the Results of Pancreatic Resections between Surgeons: A Systematic Review and Meta-Analysis of Pancreatic Resections

**DOI:** 10.1155/2015/896875

**Published:** 2015-08-17

**Authors:** Kurinchi Gurusamy, Clare Toon, Bhavisha Virendrakumar, Steve Morris, Brian Davidson

**Affiliations:** ^1^Department of Surgery, UCL Medical School, Royal Free Campus, London NW3 2PF, UK; ^2^Public Health Research Unit, West Sussex County Council, County Hall Campus, West Sussex PO19 1QT, UK; ^3^Evidence Synthesis, Sightsavers, 35 Perrymount Road, Haywards Heath, West Sussex RH16 3BW, UK; ^4^Department of Applied Health Research, UCL, London WC1E 7HB, UK

## Abstract

*Background.* Indicators of operative outcomes could be used to identify underperforming surgeons for support and training. The feasibility of identifying HPB surgeons with poor operative performance (“outliers”) based on the results of pancreatic resections is not known. *Methods.* A systematic review of Medline, Embase, and the Cochrane library was performed to identify studies on pancreatic resection including at least 100 patients and published between 2004 and 2014. Proportions that lay outside the upper 95% and 99.8% confidence intervals based on results of the systematic reviews were considered as “outliers.” *Results.* In total, 30 studies reporting on 10712 patients were eligible for inclusion in this review. The average short-term mortality after pancreatic resections was 3.1% and proportion of patients with procedure-related complications was 47.0%. None of the classification systems assessed the long-term impact of the complications on patients. The surgeon-specific mortality should be 5 times the average mortality before he or she can be identified as an outlier with 0.1% false positive rate if he or she performs 50 surgeries a year. *Conclusions.* A valid risk prognostic model and a classification system of surgical complications are necessary before meaningful comparisons of the operative performance between pancreatic surgeons can be made.

## 1. Background

Indicators of operative outcomes could be used to identify underperforming surgeons for support and training. Pancreatic resection is one of the most common major operative procedures performed by Hepato-Pancreato-Biliary (HPB) surgeons. As the procedure is complex with a high associated morbidity and mortality, it may be suitable for comparing the operative performances of HPB surgeons. The major indication for pancreatic resection is pancreatic cancer, the seventh most common cause of cancer-related mortality in the world, resulting in approximately 330 000 deaths worldwide annually [1]. Pancreatic cancer is a biologically aggressive cancer, which is relatively resistant to chemotherapy and radiotherapy and has a high rate of local and systemic recurrence [2–4]. In early pancreatic cancer (with no invasion of adjacent structures such as the superior mesenteric vein, portal vein, or superior mesenteric artery or distal metastases), surgical resection is generally considered the only treatment with the potential for long-term survival and possibility of cure in people likely to withstand major surgery. The overall five-year survival after radical resection ranges from 7% to 25% [4–9], with a median survival of 11 to 15 months [10]. With adjuvant chemotherapy, median survival after radical resection is increased and ranges between 14 and 24 months [11]. However, it should be noted that about half of the patients presenting with pancreatic cancer will have metastatic disease and one-third have locally advanced unresectable disease, leaving about 10% to 20% suitable for resection [12].

Another major indication for pancreatic resection is chronic pancreatitis, a condition associated with long-standing and progressive inflammation of the pancreas resulting in destruction and replacement of pancreatic tissue with fibrous tissue leading to exocrine pancreatic insufficiency and endocrine pancreatic insufficiency (diabetes) [13]. The annual incidence of chronic pancreatitis ranges from 1.5 to 7.9 per population of 100,000 [14–18]. The prevalence of chronic pancreatitis ranges from 17 to 49 per population of 100,000 [15, 16, 18]. The annual mortality rate attributable to chronic pancreatitis is around 1 to 4 per million people [15, 17]. There is no consensus among experts for selecting patients for surgical management but pancreatic pain and other local complications are the major indications for surgical treatment [19]. Other indications for pancreatic resection include ampullary cancers, distal common bile duct cancers, duodenal cancers, intraductal papillary mucinous neoplasm, and neuroendocrine tumours [20–23].

Pancreatic resection is in the form of pancreaticoduodenectomy for cancers of the head of the pancreas, ampullary cancers, distal common bile duct cancers, and duodenal cancers and distal pancreatectomy for cancers of the body and tail of the pancreas [24]. Pancreaticoduodenectomy involves excision of the head of the pancreas and duodenum. The two major types are the classical Whipple operation and the pylorus preserving pancreatoduodenectomy [25]. Surgical excision for chronic pancreatitis can be performed by pancreaticoduodenectomy (standard Whipple's operation or pylorus preserving pancreaticoduodenectomy) or by duodenum preserving pancreatic head resection [19, 26]. Duodenum preserving pancreatic head resection involves resection of the pancreatic head without excision of duodenum. The two major types are Beger's operation and Frey's procedure [26]. The latter involves a drainage procedure anastomosing the duct in the pancreatic remnant to the jejunum by longitudinal pancreatojejunostomy in addition to pancreatic head excision leaving behind a cuff of pancreas on the duodenal wall [26].

In general, pancreaticoduodenectomy is performed by open surgery, although laparoscopic pancreaticoduodenectomy has been reported [27]. Laparoscopic pancreatic resection is more common for distal pancreatectomy [28]. After resection of the body and tail of the pancreas, the cut surface of the pancreatic remnant (pancreatic stump) is closed using staples or sutures [29].

In England, individual surgeon's results of surgery-related complications are being published as part of the drive by NHS England to improve transparency [30, 31] and to allow patients to make informed decisions. This allows patients to identify outliers (a consultant whose clinical outcomes data lies outside the expected range given the national average) [31] and make an informed decision as to whether they would like to be treated by that particular surgeon. The feasibility of identifying HPB surgeons with poor operative performance based on the results of pancreatic resections is not known but it may be the most suited for comparison as the procedures are common and complex and are generally associated with a high morbidity and mortality. The main objectives of this research are to conduct a systematic review of the recent results of pancreatic resections so that it is possible to establish a benchmark for surgeon's performance based on international standards and to assess the feasibility of comparing the results of pancreatic resections between surgeons based on the results of the systematic review.

## 2. Methods

### 2.1. Selection of Studies

All studies that reported on pancreatic resections irrespective of whether they were pancreaticoduodenectomies or distal pancreatectomy, the reason for the pancreatic resection (cancer or benign disease), the type of access (open or laparoscopic), the type of anastomosis (pancreaticogastrostomy or pancreatojejunostomy), and the postoperative care provided to the patients were included. Only studies including at least 100 patients, published as full texts or conference abstracts in the previous 10 years from the search date (February 2014), and reporting one or more of the primary outcomes (30-day or in-hospital mortality) or secondary outcomes (12-month mortality, proportion of people with complications, number of complications, the classification system used to report the complications, operating time, and length of hospital stay) were included in the review to ensure that only the recent results on a reasonable number of patients were included in the analysis. Studies were identified by searching Medline, Embase, and the Cochrane library using the Medical Subject Headings (MeSH) search terms “pancreatectomy,” “pancreaticoduodenectomy,” and “pancreaticojejunostomy.” Equivalent free text search terms were used and equivalent search strategies were used in other databases. The search strategies are available in the Appendix. No language restrictions were applied.

Two authors (Clare Toon and Bhavisha Virendrakumar) independently screened titles and abstracts. Full texts were obtained for references that at least one author identified as potentially meeting the inclusion criteria. Further selection was made independently by two authors (Clare Toon and Bhavisha Virendrakumar) by reviewing the full texts. All differences were resolved by discussion and arbitration by another author (Kurinchi Gurusamy).

### 2.2. Data Collection

Data on patient characteristics including the demographic details, case-mix (risk prognostic models or score to take into account the different anaesthetic and surgical risks in patients), and outcomes were extracted by two authors (Clare Toon and Bhavisha Virendrakumar) independently. Foreign language articles were translated to English before data extraction. When significant overlap of patients between two or more reports was identified based on the authors, centres, and the time period, the report that contained maximum information with regard to the outcomes was included for the analysis. All differences in data extraction were resolved by discussion and arbitration by another author (Kurinchi Gurusamy).

### 2.3. Meta-Analysis

Meta-analysis was performed using StatsDirect statistical software using a random-effects model. The summary estimates with 95% confidence intervals (CI) have been reported. Heterogeneity was assessed by Higgin's *I*-square [32] and chi-square test for heterogeneity. Despite exploration of heterogeneity by various subgroup analyses including the reason for pancreatic resection (cancer versus other causes), type of resection (pancreaticoduodenectomy versus distal pancreatic resection), and method of access (laparoscopic versus open access), the data available from the studies were insufficient to allow meaningful subgroup analyses. Publication bias was assessed by funnel plot and Egger's regression test [33].

### 2.4. Assessment of Feasibility of Comparing the Operative Performance

The short-term mortality and complications which would have been attributable to an individual surgeon for a hypothetical cohort of people undergoing pancreatic resection were calculated based on the summary estimate of the meta-analysis, the lower quartile, and the upper quartile of the proportions observed for these outcomes in the systematic review, thus extrapolating the results of the meta-analysis to an average surgeon. The 95% and 99.8% confidence intervals of these outcomes were calculated using the Wilson score method with continuity correction [34] for samples sizes of 50, 100, and 200 (approximately 1 pancreatic resection a week, 2 pancreatic resections a week, and 4 pancreatic resections a week). Proportions that lay outside the upper 95% and 99.8% confidence limits were considered as outliers with a one-sided false positive rate of 2.5% and 0.1%, respectively. The 95% and 99.8% confidence limits are equivalent to the surgeon having results which are different by two standard deviations and three standard deviations from the average results expected based on the data (“the benchmark”). One-sided false positive rate was calculated since the upper limit of the confidence interval was the main interest of the study; that is, if surgeon-specific mortality and complications were lower than the confidence limits, it was not of any interest since these surgeons are better than other surgeons and there are no concerns on their operative performance.

## 3. Results

### 3.1. Search Results

**Figure 1:**
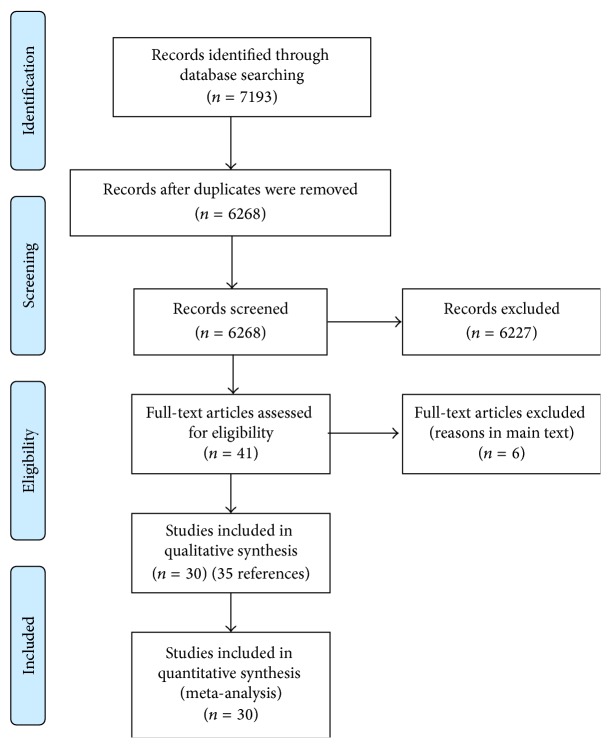
Reference flow. Reference flow showing the study selection [77].

A total of 7193 references were identified by database search. After removing duplicate citations, a total of 6268 unique references were identified. Full text was sought for 41 references [20–23, 35–71]. A total of 6 full texts were excluded (4 studies had less than 100 patients in total [66–69]; one reference was a comment on an excluded article [70]; and one study did not contain any outcomes included in this review [71]). Five references were duplicate reports of other studies or contained a significant proportion of patients included in other reports [61–65]. Data from these studies was not included in the analysis to avoid the same patients being counted multiple times. In total, 30 studies reporting on 10712 patients were eligible for inclusion in this review [20–23, 35–60]. The reference flow is shown in Figure 1.

### 3.2. Characteristics of Included Studies

The number of patients included in each study, the number and proportion of patients with malignancy, the mean age of patients, the number and proportion of female patients, and different groups within the cohort as reported by the study authors have been tabulated in Table 1. The number of patients included in each study varied from 100 to 2610 patients. Three studies included only patients with chronic pancreatitis [36, 38, 53]. One study included only patients with malignancy [37]. The remaining studies included various proportions of patients with malignancy. The mean age of patients reported in the studies ranged from 42 years to 68 years. Case-mix was assessed using surgical Apgar score (SAS) in one study [39]. None of the remaining studies reported any adjustment for case-mix. The surgical details of patients in terms of the surgeries and the surgical access included in the studies have been tabulated in Table 2. Only three studies included patients undergoing laparoscopic pancreatic resection [20, 22, 41]. It is likely that most of the patients or all the patients in the other studies underwent open pancreatic resection.

### 3.3. Outcomes

#### 3.3.1. Short-Term Mortality

**Figure 2:**
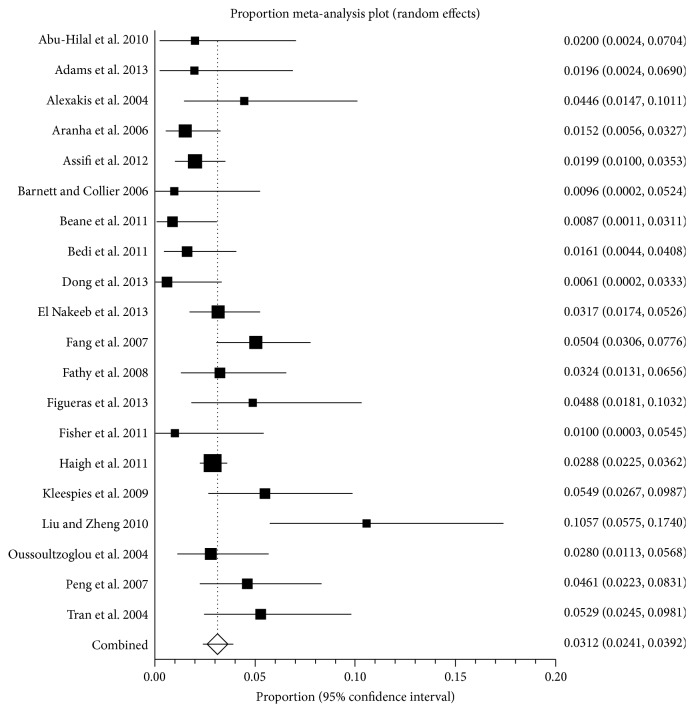
30-day or in-hospital mortality. The figure shows the forest plot of 30-day or in-hospital mortality. The mortality ranged between 0.6% and 10.6%. The average mortality by random-effects model was 3.1%.

Short-term mortality (30-day or in-hospital mortality) was reported in 21 studies including 6727 patients [21, 23, 35, 36, 38, 39, 41, 42, 44–48, 50–55, 58]. The 30-day or in-hospital mortality ranged between 0.6% and 10.6% (lower quartile = 1.6%; upper quartile = 4.7%). The mortality proportions in individual studies are shown in Figure 2. The average mortality was 3.1% (95% CI 2.4% to 3.9%; *I* ^2^ = 59.6%). There was no evidence of publication bias by Egger's regression test (*P* = 0.1866).

Depending upon the proportion of short-term mortality used (the meta-analysis summary estimate, lower quartile, or upper quartile), sample size (50, 100, or 200), and the false positive rate (2.5% versus 0.1% for a surgeon to be wrongly identified as an outlier), a surgeon will be called an outlier only when the surgeon-specific mortality is several times the average mortality (Table 3). For example, the surgeon-specific mortality should be more than 5 times the average mortality before he or she can be identified as an outlier with 0.1% false positivity rate (i.e., results lie outside three standard deviations of the average results expected from a surgeon) if he or she performs 50 surgeries a year.

#### 3.3.2. 12-Month Mortality

**Figure 3:**
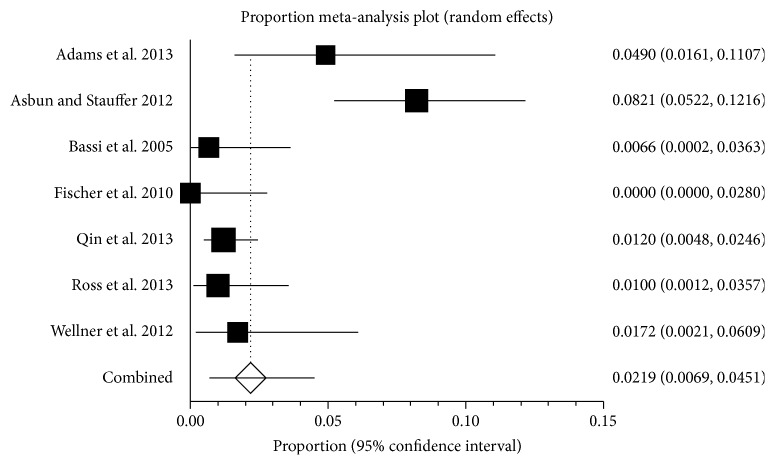
12-month mortality. The figure shows the forest plot of 12-month mortality. The mortality ranged between 0.0% and 8.2%. The average mortality by random-effects model was 2.2%.

Twelve-month mortality was reported in 7 studies including 1549 patients [22, 36, 40, 49, 56, 57, 59]. The 12-month mortality ranged between 0.0% and 8.2% (lower quartile = 0.9%; upper quartile = 3.3%). The mortality proportions in individual studies are shown in Figure 3. The average mortality was 2.2% (95% CI 0.7% to 4.5%; *I* ^2^ = 83.7%). There was no evidence of publication bias by Egger's regression test (*P* = 0.1174).

#### 3.3.3. Complications

Complications were reported variably in different studies. Five studies reported complications using the Clavien-Dindo method [72, 73] of classification of complications [20, 37, 39, 48, 60]. One study used the Accordion severity grading system [74] of classification of complications [22]. Two studies used “common terminology criteria for adverse events” system [75] of classification of complications [50, 57]. The remaining studies did not use any specific system of classification of complications.

**Figure 4:**
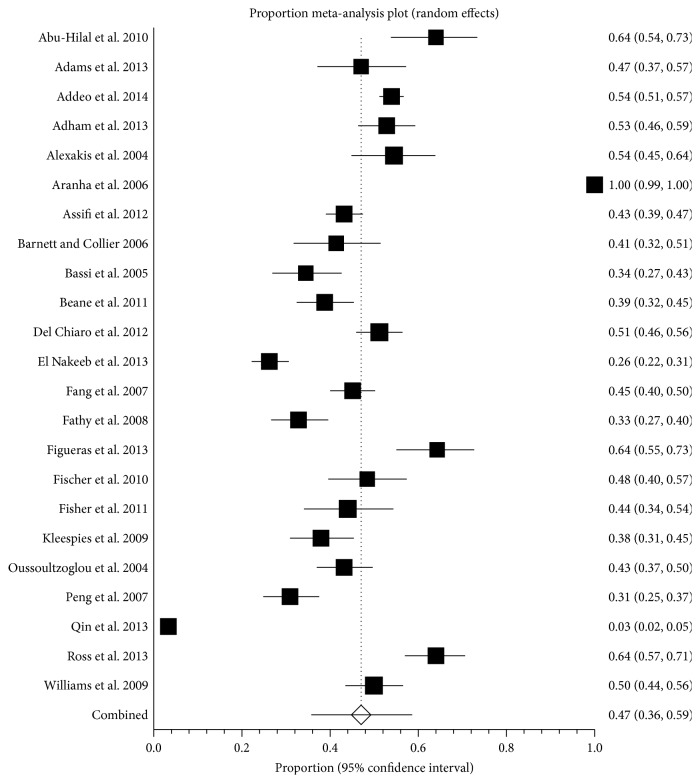
Proportion of patients with complications. The figure shows the forest plot of patients with complications. The proportion of people with complications ranged between 3.3% and 100.0%. The average proportion of complications by random-effects model was 47.0%.

The proportion of people with complications was reported in 23 studies including 6712 patients [20, 21, 23, 35–41, 43, 45–50, 52, 54–57, 60]. The proportion of people with complications ranged between 3.3% and 100.0% (lower quartile = 38.3%; upper quartile = 53.4%). The proportions of people with complications in individual studies are shown in Figure 4. The average proportion of people with complications was 47.0% (95% CI 36.0% to 59.0%; *I* ^2^ = 98.9%). There was significant publication bias as denoted by Egger's regression test (*P* = 0.0037) with the funnel plot suggesting that studies with lower complication proportions were more likely to be published.

With regard to comparing the performance of surgeons, a surgeon will be identified as an outlier with 0.1% false positive rate when the proportion of patients who develop complications following surgery by him or her is 1.4 times that of the average even if he or she performs 50 surgeries a year as shown in Table 3.

**Figure 5:**
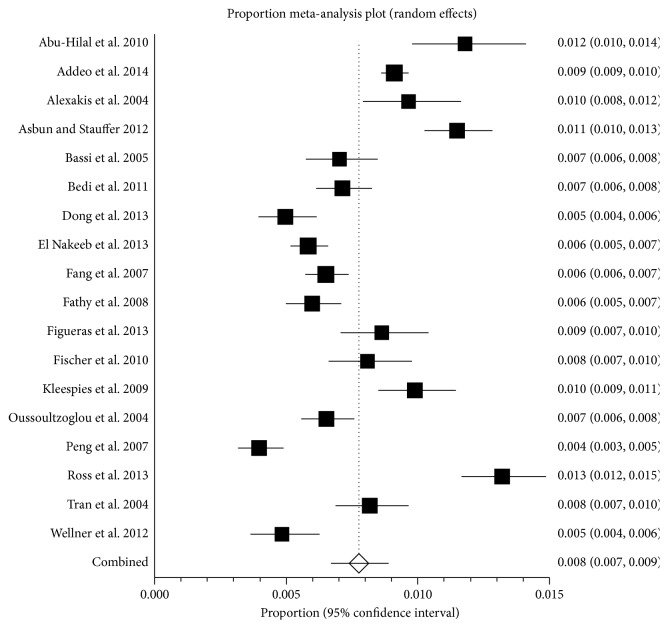
Number of complications. The figure shows the forest plot of number of complications. The number of complications per 100 patients ranged between 40 and 132. The average number of complications per 100 patients by random-effects model was 80.

The number of complications (as opposed to the proportion of people with complications) was reported in 18 studies including 4763 patients [22, 35, 37, 38, 40, 42, 44–49, 52, 54, 55, 57–59]. The number of complications per 100 patients ranged between 40 and 132 (lower quartile = 61 per 100 patients; upper quartile = 95 per 100 patients). The numbers of complications per 100 patients in individual studies are shown in Figure 5. The average number of complications per 100 patients was 80 (95% CI 70 to 90; *I* ^2^ = 94.3%). There was no evidence of publication bias by Egger's regression test (*P* = 0.4189).

#### 3.3.4. Operating Time

The average operating time was reported as mean or median in 22 studies including 5475 patients [20–22, 36, 39–41, 44–49, 52–60]. A meta-analysis was not performed because of insufficient data (i.e., mean and standard deviation were not reported adequately in many studies). The mean or median operating time in the studies ranged between 230 minutes and 492 minutes (median = 337 minutes; lower quartile = 279 minutes; upper quartile = 419 minutes).

#### 3.3.5. Hospital Stay

The average hospital stay was reported as mean or median in 24 studies including 7385 patients [20–23, 35, 36, 38, 40–43, 45–48, 50–52, 54, 55, 57–60]. As for operating time, meta-analysis was not performed for hospital stay because of insufficient data. The mean or median hospital stay in the studies ranged between 6 days and 31 days (median = 15 days; lower quartile = 11 days; upper quartile = 17 days).

## 4. Discussion

In this systematic review and meta-analysis, the recent results of pancreatic resections have been reviewed. Despite significant advances in anaesthetic and surgical techniques in the recent years, pancreatic resection remains a major surgery with significant risk of complications and mortality. The average 30-day or in-hospital mortality after pancreatic resection was approximately 3% (Figure 2) and approximately 47% of patients undergoing pancreatic resection develop one or more complications (Figure 4). However, there was significant variation in the mortality and the complications as evidenced by the *I* ^2^ values which demonstrated substantial statistical heterogeneity. The average 12-month mortality was 2.2% (Figure 3) which was less than the average 30-day mortality of 3.1%. This is not clinically possible but was observed in this systematic review because of different studies being included for the different time points. This is further evidence of heterogeneity in mortality between the studies.

One possible reason for this observed heterogeneity in the results is the inclusion of different types of surgeries in different studies. Another possible reason is that the patients included in the different studies had different comorbidities and there was variation in the technical difficulty of surgery (“case-mix”). Use of prognostic models is one of the commonly used methods to adjust for case-mix. A number of prognostic models are available for risk adjustment in pancreatic resections [76], although the accuracy of these models has not been assessed systematically. Only one of the studies included in this review considered a risk prognostic model to adjust for case-mix [39]. While the authors used surgical Apgar score as the risk prognostic model, it was not reliable in this study [39].

Prognostic models to adjust for case-mix are essential for comparative audit between specialists to ensure that surgeons are not penalised for accepting to operate on high-risk patients where there is evidence of potential patient benefit. In addition, adequate adjustment for the case-mix is necessary to allow indirect comparison of results obtained in different studies. Thus, a reliable method of adjustment for case-mix (risk prognostic model) is necessary for pancreatic resections.

With regard to complications, in addition to the types of pancreatic resections and case-mix contributing to the heterogeneity in the estimates obtained in the different studies, another reason for heterogeneity is the different methods of classifying complications.

While the mortality rate of 3% is a high perioperative mortality rate, it does not allow comparison of the surgical performance as the surgeon-specific mortality has to be more than 5 times the average mortality before the surgeon is identified as an outlier with 0.1% false positive rate if he or she performs 50 pancreatic resections a year (Table 3). Fifty pancreatic resections per year equates to an average of one resection a week and few surgeons are likely to perform more than this. Thus, using short-term mortality does not appear to be a sensitive way of comparing the performance of HPB or pancreatic surgeons. If, on the other hand, complication rates were used to compare surgeons, an outlier will be identified if the proportion of patients who develop complications following surgery by him or her is only 1.4 times the average complication rates (with 0.1% false positive rate if he or she performs 50 pancreatic resections a year). An evaluation of complication rates following pancreatic resection would therefore allow the comparison of operative performance of surgeons with a reasonable sensitivity. However, the major problem with using complications as the benchmark for assessing surgeons is that they will also depend on the case-mix of the patient cohort. In addition, none of the current classification systems for complications adequately distinguish between complications that result in permanent disability as opposed to those that do not result in permanent disability. While some of these systems include reinterventions and requirement for organ support while classifying complications [72–74], the cost implications of these individual complications to the healthcare funder are not clear. Thus, the existing systems of classification of complications which have been applied to major pancreatic surgery do not appear to be patient outcome oriented or funder oriented and cannot therefore be used as benchmark for assessing surgical performance.

Health-related quality of life (HRQoL) using a validated quality of life scale may be a suitable way of comparing the long-term outcomes of surgeons but is not sensitive enough to capture the severity of the early postoperative complications. This is because the HRQoL is usually impaired immediately after major surgery and hence those developing major complications shortly after surgery may not have a significant change from the baseline (observed in people without complications) because of the low baseline values. In addition, measurement of long-term HRQoL may necessitate additional follow-up for patients resulting in additional resource utilisation and costs. The likelihood of missing data will increase if long-term follow-up is necessary to assess the outcomes.

Current methods which have been suggested for identifying surgeons with poor operative performance are likely to miss a significant proportion of underperforming pancreatic surgeons. The results of this review are applicable to other surgeries that have similar or lower mortality such as liver resections and colorectal surgeries. Valid risk prognostic model and classification system of surgical complications (which captures long-term disability to patients and the cost implications to funder) are necessary before meaningful comparisons of the operative performance between pancreatic surgeons can be made.

## Figures and Tables

**Table 1:** Population characteristics.

Study ID	Number of patients	Cancers (percentage)	Groups^*∗*^			Numbers in each group			Average age in years	Females (percentage)
			Group 1	Group 2	Group 3	Group 1	Group 2	Group 3		
Abu-Hilal et al. 2010 [35]	100	Not reported	Feeding through percutaneous jejunostomy feeding tube	Feeding through percutaneous transgastric jejunal feeding tube	Feeding through nasojejunal feeding tube	25	32	43	67	45 (45%)

Adams et al. 2013 [36]	102	0 (0%)	Total pancreatectomy with islet autotransplant	—	—	102	—	—	42	79 (77.5%)

Addeo et al. 2014 [37]	1325	1325 (100%)	Pancreaticogastrostomy	Pancreaticojejunostomy	—	733	563	—	65	754 (56.9%)

Adham et al. 2013 [20]	242	Not reported	Drain	No drain	—	130	112	—	62	115 (47.5%)

Alexakis et al. 2004 [38]	112	0 (0%)	Opioid use prior to surgery	No opioid use prior to surgery	—	66	46	—	46	35 (31.3%)

Aranha et al. 2006 [21]	396	168 (42.4%)	Pancreaticoduodenectomy	Distal pancreatectomy with splenectomy	—	396	86	—	67	169 (42.7%)

Asbun and Stauffer 2012 [22]	268	130 (48.5%)	Open procedure	Laparoscopic procedure	—	215	53	—	66	144 (53.7%)

Assifi et al. 2012 [39]	597	Not reported	Pancreaticoduodenectomy	Suspension pancreatic duct-jejunum end-to-side continuous suture anastomosis	—	553	65	—	64	284 (47.6%)

Barnett and Collier 2006 [23]	104	85 (81.7%)	Patients undergoing pancreaticoduodenectomy	—	—	104	—	—	63	47 (45.2%)

Bassi et al. 2005 [40]	163	89 (54.6%)	Pancreaticogastrostomy	Pancreaticojejunostomy	—	69	82	—	57	56 (34.4%)

Beane et al. 2011 [41]	230	63 (27.4%)	Endoscopic ultrasound	No endoscopic ultrasound	Distal pancreatectomy	179	51	—	60	143 (62.2%)

Bedi et al. 2011 [42]	248	Not reported	Pancreaticojejunostomy	Pancreaticogastrostomy	—	126	122	—	Not reported	Not reported

Del Chiaro et al. 2012 [43]	367	Not reported	Overweight or obese	Normal weight	—	141	226	—	Not reported	Not reported

Dong et al. 2013 [44]	165	156 (94.5%)	End-to-end/end-to-side invaginated anastomosis	End-to-side mucosal anastomosis	Suspension pancreatic duct-jejunum end-to-side continuous suture anastomosis	52	48	65	51	50 (30.3%)

El Nakeeb et al. 2013 [45]	442	Not reported	Cirrhotic liver	Noncirrhotic liver	—	67	375	—	53	165 (37.3%)

Fang et al. 2007 [46]	377	319 (84.6%)	Pancreaticojejunostomy	Pancreaticogastrostomy	—	188	189	—	68	116 (30.8%)

Fathy et al. 2008 [47]	216	Not reported	Pancreaticoduodenal resection	—	—	216		—	54	85 (39.4%)

Figueras et al. 2013 [48]	130	104 (80%)	Duct-to-duct pancreaticojejunostomy	Double-layer invaginated pancreaticogastrostomy	—	58	65	—	66	42 (32.3%)

Fischer et al. 2010 [49]	209	85 (40.7%)	Acute normovolemic haemodilution	Standard intraoperative management	—	65	65	—	65	61 (29.2%)

Fisher et al. 2011 [50]	100	Not reported	Nasogastric tubes removed in the early postoperative period	Nasogastric tubes removed in the operating room at the conclusion of surgery	—	50	50	—	62	56 (56%)

Haigh et al. 2011 [51]	2610	1828 (70%)	Younger (patients aged under 70 years)	Elder (patients aged over 70 years)	—	1633	799	—	64	1330 (51%)

Kleespies et al. 2009 [52]	182	160 (87.9%)	Cattell-Warren pancreaticojejunostomy	Blumgart anastomosis	—	90	92	—	66	77 (42.3%)

Liu and Zheng 2010 [53]	123	0 (0%)	Pancreaticoduodenectomy	Duodenum preserving pancreatic head resection	—	57	66	—	Not reported	Not reported

Oussoultzoglou et al. 2004 [54]	250	175 (70%)	Pancreaticogastrostomy	Pancreaticojejunostomy	—	167	83	—	59	96 (38.4%)

Peng et al. 2007 [55]	261	194 (74.3%)	Binding pancreaticojejunostomy	Conventional pancreaticojejunostomy	—	106	111	—	52	100 (38.3%)

Qin et al. 2013 [56]	582	Not reported	Pancreaticoduodenectomy		—	582	—	—	Not reported	Not reported

Ross et al. 2013 [57]	200	108 (54%)	No perioperative transfusion	Perioperative transfusion	—	164	36	—	64	113 (56.5%)

Tran et al. 2004 [58]	170	Not reported	Standard Whipple	Pylorus preserving pancreaticoduodenectomy	—	83	87	—	63	62 (36.5%)

Wellner et al. 2012 [59]	267	121 (45.3%)	Pancreaticogastrostomy	Pancreaticojejunostomy	—	59	57	—	66	60 (22.5%)

Williams et al. 2009 [60]	174	172 (71.7%)	Normal weight	Overweight	Obese	103	71	66	65	123 (70.7%)

^*∗*^Groups according to the way the authors divided the patients.

**Table 2:** Surgery details.

Study ID	Laparoscopic^*∗*^	Open^*∗*^	Whipple	Pylorus preserving pancreaticoduodenectomy	Distal pancreatectomy
Abu-Hilal et al. 2010 [35]	Not stated	Not stated	Yes	No	No
Adams et al. 2013 [36]	Not stated	Not stated	Not stated	Not stated	Not stated
Addeo et al. 2014 [37]	Not stated	Not stated	Not stated	Yes	Not stated
Adham et al. 2013 [20]	Yes	Yes	Yes	Yes	Yes
Alexakis et al. 2004 [38]	Not stated	Not stated	Yes	Yes	No
Aranha et al. 2006 [21]	Not stated	Not stated	Yes	Yes	No
Asbun and Stauffer 2012 [22]	Yes	Yes	Yes	Yes	No
Assifi et al. 2012 [39]	Not stated	Not stated	Yes	Yes	No
Barnett and Collier 2006 [23]	Not stated	Not stated	Not stated	Not stated	Not stated
Bassi et al. 2005 [40]	Not stated	Not stated	Not stated	Yes	Not stated
Beane et al. 2011 [41]	Yes	Yes	No	No	Yes
Bedi et al. 2011 [42]	Not stated	Not stated	Yes	No	No
Del Chiaro et al. 2012 [43]	Not stated	Not stated	Not stated	Not stated	Not stated
Dong et al. 2013 [44]	Not stated	Not stated	Not stated	Not stated	Not stated
El Nakeeb et al. 2013 [45]	Not stated	Not stated	Yes	Yes	No
Fang et al. 2007 [46]	Not stated	Not stated	Yes	Yes	Not stated
Fathy et al. 2008 [47]	Not stated	Not stated	Yes	Yes	Not stated
Figueras et al. 2013 [48]	Not stated	Not stated	Yes	Yes	No
Fischer et al. 2010 [49]	Not stated	Not stated	Yes	Yes	Not stated
Fisher et al. 2011 [50]	Not stated	Not stated	Yes	Yes	Yes
Haigh et al. 2011 [51]	Not stated	Not stated	Yes	Yes	Not stated
Kleespies et al. 2009 [52]	Not stated	Not stated	Yes	Yes	Not stated
Liu and Zheng 2010 [53]	Not stated	Not stated	Not stated	Not stated	Not stated
Oussoultzoglou et al. 2004 [54]	Not stated	Not stated	Yes	Yes	Not stated
Peng et al. 2007 [55]	No	Yes	Yes	Yes	Not stated
Qin et al. 2013 [56]	Not stated	Not stated	Not stated	Not stated	Not stated
Ross et al. 2013 [57]	Not stated	Not stated	Not stated	Not stated	Not stated
Tran et al. 2004 [58]	Not stated	Not stated	Yes	Yes	No
Wellner et al. 2012 [59]	Not stated	Not stated	Yes	Yes	Not stated
Williams et al. 2009 [60]	Not stated	Not stated	Yes	Yes	Not stated

^*∗*^It is likely that the studies that do not report on whether the surgeries were performed by open or laparoscopic method are likely to include open access surgeries.

**Table 3:** Identification of outliers.

Outcome and proportion	Sample size	Outlier (2.5% false positive)	Outlier (0.1% false positive)
Mortality: meta-analysis summary (3.1%)	50	>12.2%	>16.7%
	100	>8.6%	>11.2%
	200	>6.5%	>8.1%

Mortality: lower quartile (1.6%)	50	>9.9%	>14.4%
	100	>6.4%	>9.0%
	200	>4.5%	>5.9%

Mortality: upper quartile (4.7%)	50	>14.4%	>19.0%
	100	>10.8%	>13.5%
	200	>8.6%	>10.2%

Complications: meta-analysis summary (47.0%)	50	>60.5%	>64.4%
	100	>56.7%	>59.6%
	200	>53.9%	>56.0%

Complications: lower quartile (38.3%)	50	>52.1%	>56.4%
	100	>48.1%	>51.2%
	200	>45.2%	>47.4%

Complications: upper quartile (53.4%)	50	>66.5%	>70.1%
	100	>62.9%	>65.6%
	200	>60.2%	>62.2%

## Appendix

### A. Search Date: 22 February 2014

#### A.1. Medline (OvidSP): 3764

(1) exp Pancreatectomy/
(2) (pancreatectomy or pancreatectomies).ti. or (pancreatectomy or pancreatectomies).ab.
(3) ((pancreas or pancreatic) adj (resection or resections)).ti. or ((pancreas or pancreatic) adj (resection or resections or operation or operations or surgery or surgeries)).ab.
(4) exp Pancreaticoduodenectomy/
(5) (Pancreaticoduodenectomy or pancreaticoduodenectomies or duodenopancreatectomy or duodenopancreatectomies).ti. or (Pancreaticoduodenectomy or pancreaticoduodenectomies or duodenopancreatectomy or duodenopancreatectomies).ab.
(6) ((Whipple adj (procedure or procedures or operation or operations)) or PPPD).ti. or ((Whipple adj (procedure or procedures or operation or operations or surgery or surgeries)) or PPPD).ab.
(7) exp Pancreaticojejunostomy/
(8) (pancreaticojejunostomy or pancreaticojejunostomies or pancreaticogastrostomy or pancreaticogastrostomies).ti. or (pancreaticojejunostomy or pancreaticojejunostomies or pancreaticogastrostomy or pancreaticogastrostomies).ab.
(9) (1) or (2) or (3) or (4) or (5) or (6) or (7) or (8)
(10) randomized controlled trial.pt.
(11) controlled clinical trial.pt.
(12) randomized.ab.
(13) placebo.ab.
(14) drug therapy.fs.
(15) randomly.ab.
(16) trial.ab.
(17) groups.ab.
(18) (10) or (11) or (12) or (13) or (14) or (15) or (16) or (17)
(19) exp animals/not humans.sh.
(20) (18) not (19)
(21) exp cohort studies/
(22) cohort ^*∗*^.tw.
(23) controlled clinical trial.pt.
(24) epidemiologic methods/
(25) limit (4) to yr = 1966–1989
(26) exp case-control studies/
(27) (case$ and control$).tw.
(28) (case$ and series).tw.
(29) (21) or (22) or (23) or (25) or (26) or (27) or (28)
(30) (20) or (29)
(31) (9) and (30)
(32) limit (31) to (humans and last 10 years)

#### A.2. Embase (OvidSP): 4194

(1) exp pancreas resection/
(2) (pancreatectomy or pancreatectomies).ti. or (pancreatectomy or pancreatectomies).ab.
(3) ((pancreas or pancreatic) adj (resection or resections)).ti. or ((pancreas or pancreatic) adj (resection or resections or operation or operations or surgery or surgeries)).ab.
(4) exp pancreaticoduodenectomy/
(5) (Pancreaticoduodenectomy or pancreaticoduodenectomies or duodenopancreatectomy or duodenopancreatectomies).ti. or (Pancreaticoduodenectomy or pancreaticoduodenectomies or duodenopancreatectomy or duodenopancreatectomies).ab.
(6) ((Whipple adj (procedure or procedures or operation or operations)) or PPPD).ti. or ((Whipple adj (procedure or procedures or operation or operations or surgery or surgeries)) or PPPD).ab.
(7) exp pancreaticojejunostomy/
(8) (pancreaticojejunostomy or pancreaticojejunostomies or pancreaticogastrostomy or pancreaticogastrostomies).ti. or (pancreaticojejunostomy or pancreaticojejunostomies or pancreaticogastrostomy or pancreaticogastrostomies).ab.
(9) (1) or (2) or (3) or (4) or (5) or (6) or (7) or (8)
(10) exp crossover-procedure/or exp double-blind procedure/or exp randomized controlled trial/or single-blind procedure/
(11) (((((random ^*∗*^ or factorial ^*∗*^ or crossover ^*∗*^ or cross over ^*∗*^ or cross-over ^*∗*^ or placebo ^*∗*^ or double ^*∗*^) adj blind ^*∗*^) or single ^*∗*^) adj blind ^*∗*^) or assign ^*∗*^ or allocat ^*∗*^ or volunteer ^*∗*^).af.
(12) (10) or (11)
(13) exp cohort analysis/
(14) exp longitudinal study/
(15) exp prospective study/
(16) exp follow up/
(17) cohort ^*∗*^.tw.
(18) exp case control study/
(19) (case ^*∗*^ and control ^*∗*^).tw.
(20) exp case study/
(21) (case ^*∗*^ and series).tw.
(22) (13) or (14) or (15) or (16) or (17) or (18) or (19) or (20) or (21)
(23) (12) or (22)
(24) (9) and (23)
(25) limit (24) to (human and last 10 years)

#### A.3. Cochrane: 205

(#1) MeSH descriptor: [Pancreatectomy] explode all trees
(#2) (pancreatectomy or pancreatectomies)
(#3) (pancreas or pancreatic) near (resection or resections)
(#4) MeSH descriptor: [Pancreaticoduodenectomy] explode all trees
(#5) (Pancreaticoduodenectomy or pancreaticoduodenectomies or duodenopancreatectomy or duodenopancreatectomies)
(#6) ((Whipple near (procedure or procedures or operation or operations)) or PPPD)
(#7) MeSH descriptor: [Pancreaticojejunostomy] explode all trees
(#8) (pancreaticojejunostomy or pancreaticojejunostomies or pancreaticogastrostomy or pancreaticogastrostomies)
(#9) (#1) or (#2) or (#3) or (#4) or (#5) or (#5) or (#6) or (#7) or (#8) from 2004 to 2014
